# Fas expression in memory CD8+ T cell subsets augments cellular differentiation and effector function

**DOI:** 10.1186/2051-1426-3-S2-P329

**Published:** 2015-11-04

**Authors:** Tori Yamamoto, Anthony Leonardi, Hui Liu, Ena Wang, Luca Gattinoni, Anthony Cruz, Claudia Ouyang, Richard Siegel, Nicholas Restifo, Christopher A Klebanoff

**Affiliations:** 1 Center for Cancer Research, NCI/NIH, Bethesda, MD, USA; 2 Infectious Disease and Immunogenetics Section, Department of Transfusion Medicine, Clinical Center, NIH, Bethesda, MD, USA; 3 Experimental Transplantation and Immunology Branch, NCI/NIH, Bethesda, MD, USA; 4 National Institute of Arthritis and Musculoskeletal and Skin Diseases, NIH, Bethesda, MD, USA

## 

Memory CD8^+^ T cells (T_Mem_) have the capacity to provide lifelong host protection against intracellular pathogens and cancer. Despite phenotypic and functional heterogeneity among T_Mem_, the expression of Fas — a tumor necrosis family receptor (TNFR) superfamily member conventionally known as a death receptor — is held in common among all T_Mem_ subsets across multiple species. As Fas has been shown to mediate non-death signaling in other cell types, we set out to elucidate the role of Fas signaling in defined T_Mem_ subsets, including T stem cell memory (T_SCM_), T central memory (T_CM_), and T effector memory (T_EM_). We found that augmenting Fas signaling in stimulated T_SCM_ using an oligomerized form of its ligand FasL resulted in augmented cellular differentiation and a loss in IL-2 secretion capacity. Conversely, antibody blockade (anti-FasL) of Fas signaling in T_CM_ retarded cellular differentiation both phenotypically and functionally. To genetically disentangle the pro-apoptotic and differentiation signals from Fas, we made use of a mutant Fas lacking a transmembrane cysteine residue (FasC194V) that is unable to undergo S-palmitoylation and aggregate efficiently in lipid rafts. Using transgenic mice expressing this C194V Fas construct on a Fas-deficient *lpr* background, we found that FasC194V T_Mem_ can still undergo cellular differentiation in the absence of death signaling. *In vivo*, T_Mem_ expanded with anti-FasL showed greater expansion, on-target immunity and withheld differentiation. Additionally, in a relevant syngeneic model of current human T cell immunotherapy, T_Mem_ cells expanded with anti-FasL and genetically engineered with an anti-CD19 chimeric antigen receptor (CAR) exhibited enhanced CAR expression, reduced differentiation, and augmented anti-lymphoma activity compared to controls. These studies demonstrate that Fas signaling promotes not only cell death but also T_Mem_ effector differentiation, a finding that has implications for the design and execution of T cell-based immunotherapies in patients with cancer or infectious disease.

